# Use of Twitter in Neurology: Boon or Bane?

**DOI:** 10.2196/25229

**Published:** 2021-05-14

**Authors:** Biswamohan Mishra, Monica Saini, Carolynne M Doherty, Robert D S Pitceathly, Roopa Rajan, Omar K Siddiqi, Gita Ramdharry, Ajay Asranna, Pedro Jose Tomaselli, Allan G Kermode, Jawad A Bajwa, Divyani Garg, Venugopalan Y Vishnu

**Affiliations:** 1 Department of Neurology All India Institute of Medical Sciences New Delhi India; 2 Department of Neurology National Neuroscience Institute Novena Singapore; 3 Department of Neuromuscular Diseases University College London Queen Square Institute of Neurology and The National Hospital for Neurology and Neurosurgery University College London London United Kingdom; 4 Department of Neurology Harvard Medical School Boston, MA United States; 5 Department of Neurology University of Zambia School of Medicine Lusaka Zambia; 6 Department of Neurology National Institute of Mental Health and Neurosciences Bengaluru India; 7 Department of Neurosciences and Behaviour Sciences Clinical Hospital of Ribeirão Preto University of São Paulo Ribeirão Preto Brazil; 8 Department of Neurology Perron Institute, Queen Elizabeth II Medical Centre University of Western Australia Perth Australia; 9 Department of Neurology National Neuroscience Institute King Fahad Medical City Riyadh Saudi Arabia; 10 Department of Neurology Lady Hardinge Medical College New Delhi India

**Keywords:** Twitter, neurology, tweet chats, research, tweetorials, contemporary issues

## Abstract

Twitter is a free, open access social media platform that is widely used in medicine by physicians, scientists, and patients. It provides an opportunity for advocacy, education, and collaboration. However, it is likely not utilized to its full advantage by many disciplines in medicine, and pitfalls exist in its use. In particular, there has not been a review of Twitter use and its applications in the field of neurology. This review seeks to provide an understanding of the current use of Twitter in the field of neurology to assist neurologists in engaging with this potentially powerful application to support their work.

## Introduction

Twitter is a free-to-use social networking and microblogging platform, enabling registered users to post (ie, tweet), read, and repost (ie, retweet) short messages known as tweets. Tweets are limited to 280 characters—previously 140—and users can share photos, short videos, or website URLs [1]. The limited character count is an important consideration when tweeting, and encourages succinct delivery of concepts or acts as the starting point for a conversation. Messages from profiles the user has *followed*, using their @handles, appear in a curated *feed*, which is updated with new tweets at different intervals. Though Twitter has been widely used in medicine by physicians, scientists, and patients, its application in the field of neurology remains relatively uncharted.

Here we explore the powerful potential of Twitter in promoting research, education, and health care activities in neurology to relevant stakeholders. We also examine the risks and limitations of Twitter use in a professional capacity.

## Search Strategy and Selection Criteria

References for this review were identified by searching PubMed, Google Scholar, Embase, MEDLINE, and the Twitter site between March 2006 (ie, when Twitter was founded) and July 2020 as well as searching references from relevant articles. The search terms “Twitter,” “neurology,” “journal clubs,” “tweetorials,” “tweet chats,” “misuse,” “unprofessional,” “social media,” “health care,” and “medicine” were used. The search was restricted to English-language articles. The final reference list was generated on the basis of relevance to the topics covered in this review.

## Neurology and Twitter

Twitter has become an appealing platform for the scientific community to exchange ideas, information, research insights, teaching and career achievements, and opportunities. Many academic Twitter hot spots garner a huge following (eg, #AcademicChatter, #PhDChat, #Medtwitter, @OpenAcademics, and #Epitwitter). As with other social media platforms, neurologists, neurology residents, neuroscience researchers, advocacy groups, journals, professional societies, and funding agencies have all asserted their presence in the *Twitterverse* over the past few years (Tables 1 and 2 [2-4]). Tweets addressed to neurologists are often tagged #neurotwitter, and users might post anything ranging from introductions to recently published papers from their institutions to pictures of electroencephalogram (EEG)–adorned cakes.

**Table 1:** Selected neurology journals, professional associations, and medical funding agencies with a strong Twitter presence.

Journal, professional association, or funding agency			Twitter handle		Approximate follower count			
**Journal**								
	Green Journal	@GreenJournal		44,900				
	Lancet Neurology	@TheLancetNeuro		26,900				
	American Academy of Neurology (ANN)	@AANMember		23,200				
	Brain	@Brain1878		19,800				
	Nature Reviews Neurology	@NatRevNeurol		28,100				
	Nature Neuroscience	@NatureNeuro		86,400				
	JAMA Neurology	@JAMANeuro		35,700				
	Neurology Today	@NeurologyToday		44,000				
**Association**								
	Motor Neuron Disease (MND) Association	@mndassoc		31,000				
	Stroke Association	@TheStrokeAssoc		107,000				
	Alzheimer’s Research UK	@AlzResearchUK		78,000				
	American Stroke Association	@American_Stroke		19,100				
	Brain and Life Magazine	@BrainandLifeMag		34,300				
	Dementia UK	@DementiaUK		106,200				
	International Parkinson and Movement Disorder Society	@movedisorder		7157				
	World Muscle Society	@WorldMuscleSoc		800				
	The Peripheral Nerve Society (PNS)	@PNSociety1		200				
**Funding agency**								
	National Institutes of Health (NIH)	@NIH		1,100,000				
	National Institute of Neurological Disorders and Stroke (NINDS)	@NINDSnews		9555				
	Medical Research Council (MRC)	@The_MRC		65,400				
	Indian Council of Medical Research (ICMR)	@ICMRDELHI		126,400				

**Table 2:** Examples of patient support groups for neurological disorders that have a Twitter presence.

Support group	Support group Twitter handle	Activity
The Brain Charity	@TheBrainCharity	Offers emotional support, practical help, and social activities to anyone with a neurological condition
Multiple Sclerosis Foundation	@MS_Focus [3]	Helps people with multiple sclerosis (MS) live their best lives through free programs, services, and education
Stroke Association	@TheStrokeAssoc	Supports people in rebuilding their lives after stroke with their #RebuildingLives campaign
Alzheimer’s Society	@alzheimerssoc	Provides information and support for people affected by dementia (#UnitedAgainstDementia)
FSHD Society	@FSHDSociety [4]	A research-focused patient organization for facioscapulohumeral muscular dystrophy (FSHD)
Amyloidosis Support Groups	@AmyloidosisSupp	Official Twitter account of Amyloidosis Support Groups; dedicated to the support of amyloidosis patients, caregivers, and former caregivers
Parkinson’s Foundation	@ParkinsonDotOrg	Strives to make life better for people with Parkinson disease through expert care and research
FND Action	@FNDAction	A charity organization for raising awareness of functional neurological disorders (FNDs) [2], including nonepileptic attack disorder (#NEAD), and supporting those diagnosed with FNDs (#action4FND)
Muscular Dystrophy Association	@MDAorg	Strives to transform the lives of people affected by muscular dystrophy (MD), amyotrophic lateral sclerosis, and related neuromuscular diseases through innovations in science and in care
Friedreich's Ataxia Research Alliance	@CureFA_org	Works to direct and focus the resources and relationships needed to treat and cure Friedreich’s Ataxia (FA)
Hereditary Neuropathy Foundation	@CMTNeuropathy	Supports patients and families with updated relevant information and is dedicated to funding Charcot-Marie-Tooth disease (CMT)–related research

## Promoting Research

### Enhance Research Visibility and Recruitment

Twitter hashtags (ie, #) help connect people with topics of interest. By following specific profiles and hashtags and reading the associated threads, one can quickly establish an overview of the most up-to-date research activities, clinical advances, and future perspectives. This effect is particularly apparent in rare diseases, where following key handles (ie, @) and hashtags ensures that researchers, physicians, and patients can establish connections; for example, #ALSResearch (amyotrophic lateral sclerosis, ALS), @Phelan_McDermid and #PMSF (Phelan McDermid Syndrome Foundation), @thetinman_org (help and awareness for stiff-person syndrome), @yaya4HL (Yaya Foundation for 4H leukodystrophy, 4HL), @fragilexsyndrom, and numerous others. Such social media cross talk is often helpful in building clinical registries and biorepositories and may facilitate higher-quality clinical and translational research.

Twitter can help promote the visibility of clinical trials, enhancing both recruitment and inclusivity via links to enrollment websites [5]. Some such examples are as follows:

1. The Emergency Laparotomy and Frailty (ELF) study achieved a target enrollment of 500 participants by utilizing a Twitter handle (@ELFStudy) and eye-catching logos on its Twitter profile [6].
2. The AVERT DOSE trial (@AVERTDOSEtrial) posts regular updates on their work evaluating rehabilitation after stroke via their Twitter profile [7], ensuring sustained visibility.
3. Novartis is recruiting participants with advanced malignancies for US clinical trials, and its Twitter profile (@NovartisOncCT) shares the following link for potential participants to join: “http://bit.ly/2LhfJIk! #ClinicalTrialsSM” [8].
4. Wasilewski et al, in the context of a caregiver study, conducted a secondary analysis of their Twitter recruitment and found that out of the 71 caregivers, 27 were recruited via Twitter. General recruitment tweets were most frequently shared by users [9].

In a study by Sedrak et al, while the majority of the tweets (86.3%) had embedded links to news articles, one tweet led to a patient recruitment site (ie, ClinicalTrials.gov; row 7 of the second table of the study) [5]. Harnessing Twitter in this way can result in novel and larger networks.

### Facilitate International Connections, Networks, and Visibility

Twitter enables users from around the world to interact with individuals at the forefront of academic research or clinical practice. This interaction may be via tweets, comments, or both—an engagement that might not otherwise have been possible. Conference organizers can also enhance the influence of conference-related discussions beyond those attending in person through social media, including Twitter [10]. Following the people you met at a conference can also be a convenient way of light-touch networking long after a conference has ended. Some fellowship training programs use Twitter to advertise; in the United States, neurosurgery programs have the highest social media presence compared to neurology and neuroradiology [11], especially in terms of advertising residency programs [12]. Since the onset of the COVID-19 pandemic, there has been at least a 216% (from n=24 to n=76) increase in the number of neurology residency accounts on Twitter, as noted by Zelikovich et al in a blog post in Neurology [13].

Within specialties, there are focused spaces for discussion on specific topics or diseases—a global connection for users with a small area of shared interest. For instance, @Microbleeds discusses microbleed research and @ScienceofPD highlights Parkinson disease research [14]. Many also use Twitter to disseminate collaborative subspecialty education, such as neuropathology and neuroradiology, to broader audiences.

### Rapid Communication of Research

Twitter provides a platform where opinions, queries, and comments on recently published research can be posted, and reacted to, in real time facilitating easy access for clinical neurologists to recent advances. Examples of highly followed journals and societies can be seen in Table 1. Connecting clinicians and researchers is vital, as research data with translational potential may be helpful in management decisions. Moreover, input from physicians may prompt research in novel and impactful fields. Patient or public comments on research topics may also provide new perspectives and relevant research questions. Thus, Twitter can facilitate mutually beneficial, multichannel communication [15].

### Capturing a Wider Audience, Faster: #Power

Twitter facilitates patient involvement in care and has the capacity to remove barriers to understanding complex medical conditions through sharing information. By using Twitter, a patient can connect to support groups pertaining to his or her illness, such as the Motor Neuron Disease (MND) Association (@mndassoc) [16] and Parkinson’s Foundation (@ParkinsonDotOrg) [17]. Patients may interact with others who have the same disease, share experiences, and discuss treatment facilities and trials, advances in disease management, prevention, and potential sources of financial support, all of which enhance patient care. Twitter also provides a forum for all parties vested in health care to interact, including physicians, allied health care providers, patients, carers, advocacy groups, policy makers, and the pharmaceutical industry. Tagging using hashtags (ie, #) helps identify and, therefore, rapidly disseminate information to others, creating a mechanism for online multidisciplinary and interdisciplinary feedback, which may improve coordination among all relevant health care stakeholders [18].

### Role as a Search Engine: Hashtags and Handles

Twitter allows for focused reading on a particular topic of interest by providing links to research articles. For instance, someone interested in clinical genetics can follow #GeneTherapy, browse through tweets, and obtain links to pertinent websites, such as the following tweet:

Standardizing Cell and #GeneTherapy Remains Bioindustry Goal -- Please Read: http://ow.ly/3fJu50AQgLP; @AVROBIO about characterizing stem cells for gene therapy.

Individuals who have or research a specific disease can keep up to date at the level of the individual gene; for example, the inherited neuropathy gene PMP22 has its own hashtag: #PMP22.

### Conference Updates and Participation

Conference updates are shared through individual Twitter handles (eg, @SfN2020 and @abnconf) and via broader platforms using hashtags (eg, #BBIConf2018, #TheNeuroNetwork, #NeuroConference, and #AANAM). For future conferences, links can be utilized for abstract submission (eg, #Neurology2021), resulting in broader coverage and reach. It is possible to share the latest research presented during and after a conference, increasing accessibility to those unable to travel and attend in person. Consider a neurologist at a conference who replies to a tweet from @GreenJournal; others may respond in real time with critiques and may share related papers, real-life experiences, and links to related conference presentations or other research. This intertwining of experience, expertise, and communication has the potential to advance the reach of health care delivery systems. Virtual conferences have found renewed popularity in the context of the COVID-19 pandemic and benefit from advertising via Twitter; for instance, @WorldStrokOrg recently tweeted the following:

#ESOWSO2020 will run as a fully virtual event from 7-9 November. Virtual sessions will allow live interaction and will be available on-demand in the weeks following the Conference.

Another example from the Movement Disorder Society (MDS) Twitter handle @movedisorder is as follows:

New in #MDSMovingAlong: Looking Forward to the First MDS Virtual Congress. Read more from the MDS Virtual Congress Task Force on plans for this year's big event. There is still time to register at no charge before it begins on September 11. #MDSCongress.

### Platform for Journal Club and Research Review

Journal clubs on Twitter are an emerging alternative to in-person journal clubs, which are limited by time or geographical location. Twitter provides accessibility and flexibility to involve people globally, without the added pressure of hierarchy, ensuring a broader audience. For example, #NCSTJC is the Twitter hashtag for the Neurocritical Care Society’s journal club. Similar to an in-person journal club, at a Twitter journal club, a paper is selected, sometimes using crowdsourcing to ascertain participant interest. At a specific time, participants log on to Twitter, and there may be a facilitator similar to an in-person setting. Preselected questions and polls may be posted via handles or hashtags to the participants. Though there is potential for textual discussion, it is limited by the 280-character limit, unless a thread is used [19].

Traditionally, journals provide a restricted and moderated platform for research review and comments on recently published data. Twitter provides a more open platform for instant, multidimensional feedback on research quality and validity. On an individual level, reading through such comments may help broaden knowledge and enhance analytical skills. On a larger canvas, it may identify research flaws and knowledge gaps, ultimately leading to better designed and focused research. One can even seek comments from a particular organization or individual using their Twitter handle. Furthermore, there is a distinct advantage of obtaining varied views across the globe for one’s research, rather than the tunnel vision that can exist within smaller communities.

### An Alternative to Publishing Powerhouses

Academics and the public can be biased toward publications in high-impact journals, which may result in excellent research work that is published in less “prestigious” journals, thereby gaining less visibility. Social media outlets, such as Twitter, offer individuals and organizations such as universities a platform to publicize their work. However, expression of one’s expertise and research work through social networking sites, instead of through conventional platforms like journals, can be considered as less thorough or pertinent [20].

## Education and Learning

### Case Discussions

**Figure 1:**
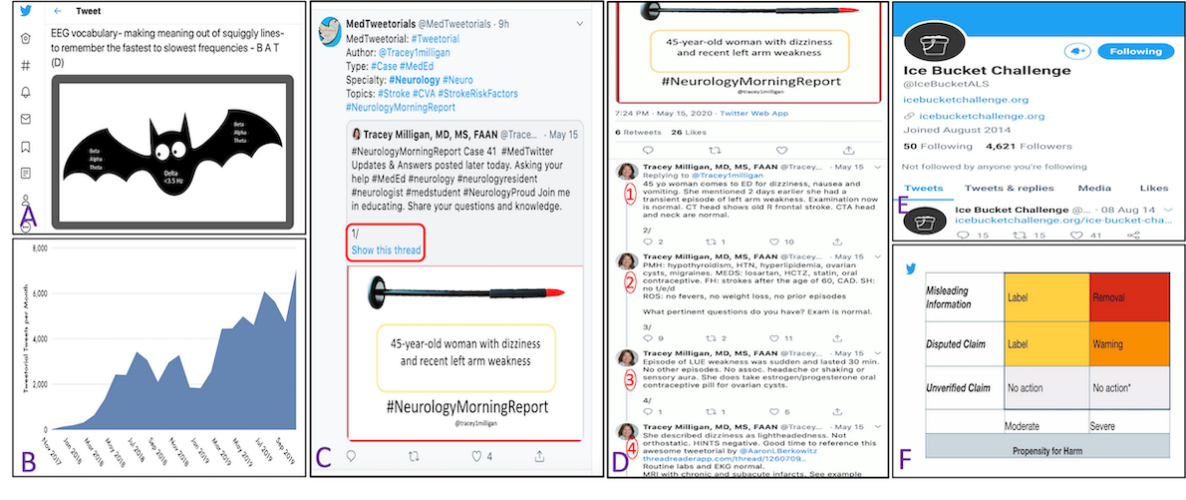
Examples of various aspects of Twitter that can be applied to neurologists. A. A representative tweet depicting a pictorial mnemonic on how to remember electroencephalogram (EEG) wave frequencies shared on Twitter. B. Monthly volume of tweetorials showing exponential growth; data were taken from Healthcare Social Graph using keyword and hashtag versions of tweetorials from November 2017 to October 2019. C and D. Examples of the use of tweetorials in neurology. C. Tracey Milligan shares an interesting clinical scenario and clicking on "show this thread" (red rectangle) opened up all the discussions pertinent to this case (D, 1), numbered here as 1, 2, 3, 4, and so on. E. A snapshot of the Ice Bucket Challenge movement on Twitter that was started to garner donations for amyotrophic lateral sclerosis (ALS) research. F. Brightly colored labels that Twitter is contemplating adding directly beneath lies and misinformation posted by users.

Twitter has opened up new dimensions to education and learning. For example, Tracy Milligan (@Tracey1milligan), through #NeurologyMorningReport [21], shares clinically relevant neurology cases via interactive, stepwise reasoning. Twitter provides a platform to tweet bite-sized informative pearls, and by the virtue of the opportunity for interaction, the chance to learn from each other’s varied clinical expertise and experience. Short, snappy, and humorous posts (eg, picture of a bat as a tool to remember EEG wave frequencies; Figure 1, A) result in a fun-filled learning experience. Thus, Twitter enhances traditional teaching by making it more interactive, exciting, and practical.

### Tweetorials

Developed in late 2017 by Mike Thompson and Vinay Prasad, *tweetorials* have seen explosive growth (Figure 1, B) [22] and have recently been accepted by the New England Journal of Medicine as a type of publication under the handle @MedTweetorials [23]. A tweetorial is a series of educational tweets posted in quick succession or a long or extended tweet, made by stringing together a series (ie, thread) of tweets [24]. Stringing together tweets, or composing a thread, allows the educator to get around the 280-character limit per tweet [25]. Because they are typically *tagged* with a keyword or hashtag and the tweets numbered by the convention 1/n, tweetorials are easy to retrieve (Figure 1, C and D). As clinical reasoning forms the backbone of neurology teaching, the structure of tweetorials has become immensely popular. For example, Crystal Yeo (@CrystalYeoMDPhD) shares tutorials on neuromuscular conditions as tweetorials.

### Tweet Chats

Tweet chats are public Twitter conversations around a unique hashtag. This hashtag allows one to track and participate in discussions as well as to connect with health care personnel and laypersons with specific interests [26]. For instance, the World Stroke Organization’s (@WorldStrokeOrg) campaign through tweet chats on COVID-19 helped spread much-needed awareness on challenges and workflow modifications during the pandemic. A representative tweet is as follows:

In the meantime, mark your calendar for the next #WSOwebinar on August 18: #Stroke Guidelines in the setting of #COVID19 with @patrice_lindsay, @sheilambrasiland Jeyaraj Pandian, @gsaposnik @neurologija @WorldStrokeEd @IntJStroke @WStrokeCampaign @BelsonSarah.

Recently, NeurologyLive and the Women Neurologists Group hosted a tweet chat on mental health in neurology, with topics addressing mental health issues and available resources from the clinician’s perspective; relevant tweets were tagged with #MindMoments. Recent tweets concerning #MindMoments have generated a staggering 1.1 million impressions [27].

### Learning and Training Opportunities

Twitter can be used to advertise and access training opportunities across all aspects of neuroscience. Examples include @NeurologistJobs, @ABNTrainees, @GetNeurologJobs, and, for the US match system, @NMatch2021. Scholars can identify and connect to potential mentors. Users of the #PhDChat network [28], created by UK doctoral students (>12,000 followers), offer advice on identifying best-fit training programs, exchange resources, and extend support via sharing experiences in academia. During the COVID-19 pandemic, PhD writing rooms, such as those hosted by @PhDForum, have evolved to help candidates focus. Curating one’s feed by following those providing relevant material can result in daily educational snippets with minimal effort. Some notable examples are as follows: (1) case- and image-based discussion on EEG hosted by Rebecca Fasano, @RebeccaFasanoMD (eg, Emory EEG Case of the Day #79 [29]); (2) @TheNotoriousEEG hosted by M Scott Perry MD [30]; (3) @ClinicalNeuroph [31], which provides news from Clinical Neurophysiology, Clinical Neurophysiology Practice, and International Federation of Clinical Neurophysiology; and (4) @MDedgeNeurology [32], which gives an overview of the most recent research and clinical developments in neurology and neuroscience.

### Workplace and Professional Concerns

Work-life balance and healthy working environments are prioritized by health care institutions. Recent studies have shown burnout to be very common among all neurology practice settings and subspecialties, with one study reporting that 60% of respondents had at least one symptom of burnout [33], and another study reporting burnout to be as high as 73% and 55% among neurology residents and fellows, respectively [34]. A study on Canadian physicians reported that more than a quarter of the physicians had mental health concerns, mostly depressed mood [35]. However, sharing of professional or workplace challenges is not always straightforward and should be approached with careful judgement and with the social media policies of relevant organizations in mind. Twitter provides a platform for individuals to connect with others with shared experiences or concerns and enables discussion that is centered on coping strategies and suggestions; the platform can act as an online *professional support group* of sorts. Examples include #BlackInNeuro, @WNGtweets (Women Neurologists Group), @LGBT_PsychNeuro, and others. Such groups have the potential to reduce isolation and enhance connectivity and visibility, and act as one of the spaces for people to discuss how to best manage the inherent challenges of the working and academic worlds.

## Community Health Care–Related Activities

### Support Groups

Many community-centric health care activities are shared through the Twitterverse. Support groups for a variety of common and, importantly, rare neurological disorders enable relationships beyond the traditional physical space (Table 2).

### Awareness Campaigns

Public awareness campaigns that use Twitter can reach a broader audience within a shorter time frame than more traditional advertising platforms. For instance, the ALS Ice Bucket Challenge (@IceBucketALS) [36] was conceived to promote awareness of ALS and to encourage donations to research. It went on to become *viral* [37] on social media during July and August 2014 (Figure 1, E), resulting in donations of US $227.6 million for ALS research in 2014 and the subsequent 4 years [38].

A study conducted in 2011 uncovered the stigma associated with epilepsy on Twitter, as 41% of tweets related to “seizure” were seen to be derogatory in nature [39]. Similar studies have also noted the use of pejorative tweets referring to psychogenic nonepileptic seizures, even by professionals [40]. Sirven et al called for a Twitter revolution to alter the way in which we perceive seizures [41]. Today, many epilepsy patient advocacy groups like the Epilepsy Foundation of America (@EpilepsyFdn; >34,000 followers) and Epilepsy Action (@epilepsyaction; >28,000 followers) regularly tweet content aiming to raise public awareness and knowledge of seizures. Only a few years later in their 2017 analysis, Meng et al found that within epilepsy and seizure accounts, verified foundations and support groups had the greatest number of accounts and users on Twitter and Facebook, with 48% of their posts aiming to provide information or to dispel common misconceptions and stigma surrounding epilepsy [42].

### Patients as a Part of Focus Groups

Twitter has been adopted by patient-focused groups (eg, NeuroImmunology Club: @NeuroImmunology [43]; MuscularDystrophyUK: @MDUK_News [44]; and Sign Against Stroke: @signagnststroke [45]) for enabling interactions between clinicians and patients, using tweet replies, and following particular handles or hashtags. This allows patients suffering from a particular illness to directly connect with their clinicians and get clarification on their illness and further treatment prospects. In the longer term, this primarily addresses patients’ main concerns but also improves the clinical community’s awareness of understanding of the disease and its management, thus bridging critical care gaps.

## Pitfalls: Ethical and Legal Concerns

### Overview

The legal and ethical issues surrounding Twitter are no less complex than those affecting the internet in general. Currently, Twitter takes some precautions in identifying the authenticity of individual users. If one chooses to make bad decisions with the information they are sharing, such as defamatory, harassing, malicious, menacing, deceptive, impersonating, or threatening tweets, they may be held liable for legal action [46]. However, regulations remain porous. Many professional bodies have issued formal guidance, such as the General Medical Council ethical guidance in the United Kingdom, Twitter Guidelines & Best Practices by the US Centers for Disease Control and Prevention, Twitter Best Practices & Tips for Physicians by Johns Hopkins Medicine, and numerous others. Some critical concerns are discussed in the following sections.

### Misrepresentation of Credentials

Individuals posing as physicians may post information that tends to be taken at face value by an unsuspecting user.

### Misinformation

Misleading messages may be widely retweeted and, hence, gain traction. No structured system currently exists to filter out misinformation on Twitter, potentially allowing inaccurately informed persons, nonprofessionals, and those with commercial interests to frame their views as facts. This can pose serious problems for trainees and patients alike, who may struggle to identify genuine and credible posts. Various groups may use the same hashtag, which confuses hashtag followers. Individuals or organizations may use a hashtag to either spread misinformation or to “bog down” a hashtag with irrelevant or unhelpful posts, which makes it harder to find the desired posts. In the nascent stages of the COVID-19 pandemic, there was a “flood of misinformation” [47] on Twitter, such that the World Health Organization termed it a “massive infodemic” [48].

Shahi et al undertook an exploratory study on COVID-19 misinformation on Twitter. Analysis of 1500 tweets relating to 1274 false and 276 partially false claims revealed that verified Twitter handles, including organizations and celebrities, were also involved in either creating or spreading the misinformation via new tweets or retweets, respectively. They also noted that false claims propagated faster than partially false claims (χ^2^_3_=10.2; *P*<.001; N=1500) [49]. Controversial posts were taken down only after large-scale objections [2,50]. In October 2020, Twitter removed a tweet by Scott Atlas, a controversial US scientist, in which he had wrongly stated that masks fail to protect against coronavirus: “Masks work? NO” [51]. In the same month, then–US President Donald Trump tweeted that the United States had “learned to live with” flu season, “just like we are learning to live with Covid, in most populations far less lethal!!!” Twitter added a caveat the same message with a warning about “spreading misleading and potentially harmful information” [52].

Twitter is experimenting with adding brightly colored labels directly beneath lies or misinformation posted by famous users (Figure 1, F), in addition to a community reports feature where inaccurate and misleading information posted by public figures is corrected directly beneath a tweet by verified fact checkers and journalists. While this may benefit the medical community [53], it may be worthwhile to explore strategies that favor dissemination of evidence-based information.

### Commercial Influences

Twitter posts with biased information may be promoted by parties with commercial interests. During the COVID-19 infodemic, the US Food and Drug Administration had to issue warnings to individuals to refrain from promoting or selling colloidal silver on social media profiles [54]. Twitter may be potentially misused by individuals and companies peddling unproven, investigational, and controversial medications, and by organizers of predatory conferences [55].

In June 2020, an Indian-based Ayurveda pharmacy claimed that it had discovered a potential cure for COVID-19, with an “80 per cent cure rate” [56]. A tweet from the chief executive officer of the pharmacy, apparently suggesting that it had obtained approval from the Ministry of AYUSH (Ayurveda, Yoga and Naturopathy, Unani, Siddha, and Homeopathy) for the drug as a “cure for COVID-19,” went viral on Twitter [57-60]. The combined drug preparation and its individual components together recorded sales of over 85 lakh units within 4 months of its launch, garnering an estimated 241 crore Indian rupees (~US $32.5 million) for the pharmacy [61]. These record sales occurred despite the fact that soon after its launch, the Ministry of AYUSH ordered the Ayurveda pharmacy to stop advertising the product as a COVID-19 cure due to the absence of appropriate clinical trial data to back its claim [61].

Similarly, predatory conferences aggressively solicit conference abstract submissions [62] and combine broad topics from multiple disciplines to cast a larger net and bring more physicians into their fold [63]. Social media, including Twitter, allows them easy access to a broad audience. Cress et al collated a list of confirmed predatory conference organizers [63]. Individuals often share their experience of receiving invitations for predatory conferences on Twitter; for example, Wim Crusio, the editor-in-chief of the journal Behavioral and Brain Functions (@WimCrusio) once shared the following [64]:

Just got an invitation for “Neurology 2019”, a world-wide meeting that attracted no less than 60 participants last year in Paris! This gem is organized by @Euroscicon. #Avoid #PredatoryConferences #FakeConferences.

Strategies to avoid such commercial misuse may include identifying and calling out suspicious activity or insisting on *identifier* hashtags.

### Exposure Bias

It is possible to be highly influenced by a small group of opinions. Users should take time to consider whether what they see is unbiased, as algorithms suggest potential profiles of interest and posts based on the interests users have already demonstrated. Twitter is an enviable educational resource, but users must always ensure that their practice is appropriately evidence based.

### Patient Confidentiality Breaches and Privacy Concerns

Twitter provides easy access to potential *research participant* data sets, which raises some concerns. In one study, 60% of Twitter users surveyed were unaware that publicly available tweets can be used for research, and 65% felt researchers should not be able to use public tweets without user permission [65-67]. There are numerous examples of patient-identifiable information being inadvertently or inappropriately shared by professionals [68].

### Unprofessional Behavior

Previous studies on verified medical Twitter users have reported a worrying trend of “unprofessional” tweets, including patient privacy violations, profanity, sexually explicit content, discriminatory statements, inaccurate information, and self-promotion [69]. While most of the violations were clearly not in keeping with a professional code of conduct, the interpretation of unprofessional was variable. Recently, a paper called *Prevalence of unprofessional social media content among young vascular surgeons* was retracted by the Journal of Vascular Surgery [70]. The researchers classified “pictures in a bikini, posted on Twitter” as unprofessional, leading to a backlash from the scientific community that was centered upon interpretation and boundaries of unprofessionalism. While some professional bodies have published ethical guidelines for doctors’ use of social media, the field remains largely unregulated [71,72]. Twitter users should keep in mind that individuals have been dismissed from their professional positions as their tweets were deemed as “unprofessional behavior” by their institutions [73].

### Breach of Intellectual Property

Sharing of prepublished content, speaker slides, and pictures taken during lectures beg the question of whether this may qualify as a breach of intellectual property. For users, it may be best to ask for permission from the primary source prior to posting information on Twitter.

### Trolling

Trolling refers to deliberate acts of making controversial comments in order to provoke internet users to respond emotionally. Trolls have used tweets to target patients with photosensitive epilepsy with flashing images intended to provoke seizures in the recent past [74,75]. Before posting, users should consider whether their posts might be considered provocative.

### Confusing Hashtag Use

Various groups may use the same hashtag and confuse hashtag followers. People may also use a hashtag incorrectly or to spread false information. In addition, people may bog down a hashtag with many posts that are not necessarily relevant or helpful. All of these make it harder to find the desired posts.

## Tips for Using Twitter

There are numerous other ethical concerns associated with physicians’ use of Twitter. One critical question is, “Who will regulate its use and how could regulations be enforced?” While Twitter has initiated, and will initiate, changes based on real-life experiences, the onus of patient confidentiality and professionalism lies with individual users. The challenges can be much higher in countries with lower English literacy, since the vast majority of information in the aforementioned resources is presented in English. The issue of language translation should be considered, as the message could be lost or transformed by translation. With this in mind, we propose some tips for using Twitter in Textbox 1.

**Tips for Twitter use in order to maintain a healthy, informative, and interactive Twitter profile.**

*How should I use Twitter?:*

1. Consider operating separate personal and professional accounts.
2. Make an honest, representative Twitter profile. Identities are periodically verified by Twitter.
3. Before sharing or retweeting, check the authenticity of the information and source. If you should reference another user’s work or tweet, use the retweet function and add a comment or use the relevant hashtag or handle to credit them. Alternatively, provide a reference or a URL to the source material.
4. Healthy critique is positive. Even if offence is not intended, comments that could be interpreted as bullying or discriminatory are ill advised. Consider whether foul language is truly necessary.
5. Educate yourself with the social media guidelines of your institution and strictly adhere to them. Remember that employers often review social media accounts—never tweet anything that could affect your professional standing or employability. In case of complaints and discrepancies, employers, regulatory bodies, or legal agencies can become involved.
6. Use meaningful hashtags. Follow a large number of other accounts and follow your followers back to maximize the number of people who see your tweets.
7. Proudly share your work. If people learn from you, they may like, follow, or retweet you.
8. When following, posting, and retweeting, endeavor to show good judgment and consider why you are saying what you are—ensure it reflects well on you and your colleagues.
9. Carefully choose the people and groups you follow or share information with. Review the privacy settings to regulate who can view your posts.
10. If you are a victim of targeted harassment or abuse, unfollow and end any communication with that user. If the behavior continues, it is recommended to block the user and report them to Twitter.

*How to maintain social media professionalism:*

1. Avoid advertising.
2. Avoid sharing any patient-identifiable information; even if anonymized, ensure you have the proper consent.
3. Demonstrate good judgement—if you should not say it in your workplace, it should remain untweeted. Avoid posting and sharing misinformation, racist or discriminatory statements, or unpleasant or explicit content. Think carefully before tweeting: if necessary, compose your tweet and review it after a short period before posting.

## Conclusions

The Twitterverse is a vast and exponentially growing expanse of information. Neurologists have taken to Twitter to educate, promote research, share information rapidly, and reach a broader potential global audience. Twitter has added a new dimension to learning and education in neurology in a practical and interactive manner. Multichannel interactions have furthered improvements in patient care and other health care–related activities. This has been particularly beneficial in promoting, furthering, and communicating research. In addition, there is enhanced support available to patients, particularly in the fields of rare and orphan diseases. However, the pros of the Twitterverse must be balanced with the potential risks, which are common to all social media platforms. Greater reach means that misinformation and promotion of vested interests can be too easily and widely disseminated. Unregulated use can result in inappropriate and unprofessional conduct whether intentional or not, highlighting the urgent need for designing and implementing institutional, national, or specialty-specific ethical and legal guidelines on appropriate social media use. Nonetheless, with judicious use, the benefits of Twitter for a neurologist or neuroscientist outweigh the risks; a graphical rendering is provided in Figure S1 in Multimedia Appendix 1.

## Appendix

Multimedia Appendix 1 A graphical rendering of pros and cons of using Twitter from a neurologist's perspective.

## Abbreviations

4HL: 4H leukodystrophy
ALS: amyotrophic lateral sclerosis
AYUSH: Ayurveda, Yoga and Naturopathy, Unani, Siddha, and Homeopathy
EEG: electroencephalogram
ELF: Emergency Laparotomy and Frailty
MDS: Movement Disorder Society
MND: motor neuron disease
PMSF: Phelan McDermid Syndrome Foundation
