# Supplementary figures and images for: Use of Twitter in Neurology: Boon or Bane?
Source: J Med Internet Res. 2021 May 14;23(5):e25229. doi: 10.2196/25229 (PMC8164119; doi:10.2196/25229)

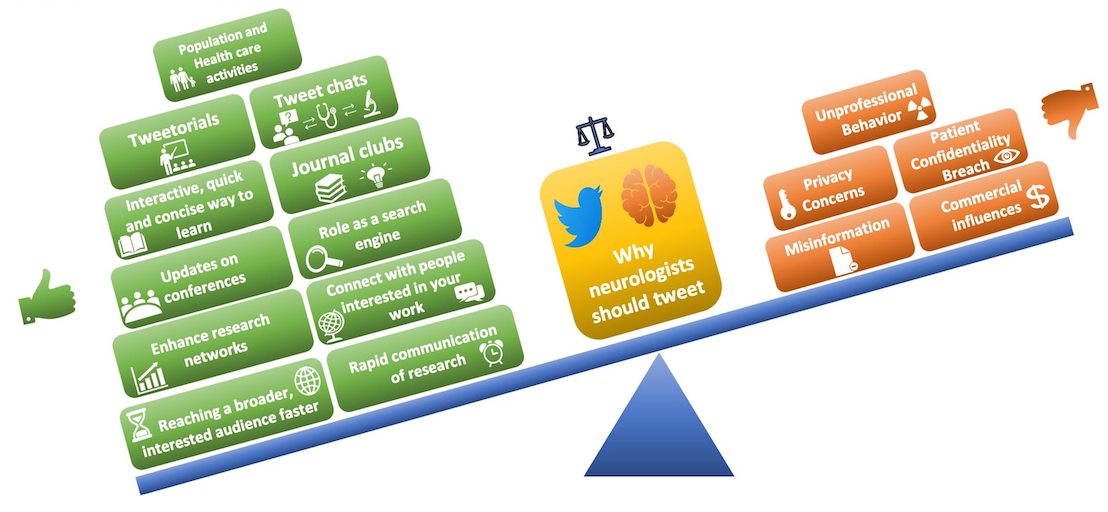

Supplement: Multimedia Appendix 1 [file jmir_v23i5e25229_app1.png]
